# Fully Ion Implanted Normally-Off GaN DMOSFETs with ALD-Al_2_O_3_ Gate Dielectrics

**DOI:** 10.3390/ma12050689

**Published:** 2019-02-26

**Authors:** Michitaka Yoshino, Yuto Ando, Manato Deki, Toru Toyabe, Kazuo Kuriyama, Yoshio Honda, Tomoaki Nishimura, Hiroshi Amano, Tetsu Kachi, Tohru Nakamura

**Affiliations:** 1 Research Center of Ion Beam Technology, Hosei University, Tokyo 184-8584, Japan; kuri@hosei.ac.jp (K.K.); t-nishi@hosei.ac.jp (T.N.); 2 Department of Electrical Engineering and Computer Science, Nagoya University, Nagoya 464-8601, Japan; yuuto_a@nuee.nagoya-u.ac.jp (Y.A.); amano@nuee.nagoya-u.ac.jp (H.A.); 3 Institute of Materials and Systems for Sustainability (IMaSS), Nagoya University, Nagoya 464-8603, Japan; deki@nuee.nagoya-u.ac.jp (M.D.); honda@nagoya-u.jp (Y.H.); kachi@imass.nagoya-u.ac.jp (T.K.); tohru@hosei.ac.jp (T.N.); 4 Toyo University, Kawagoe 350-8585, Japan; tttoyabe@gmail.com; 5 Research Center for Micro-Nano Technology, Hosei University, Tokyo 184-0003, Japan

**Keywords:** GaN, ion implantation, Mg, MOSFET, Si

## Abstract

A normally-off GaN double-implanted vertical MOSFET (DMOSFET) with an atomic layer deposition (ALD)-Al_2_O_3_ gate dielectric film on a free-standing GaN substrate fabricated by triple ion implantation is presented. The DMOSFET was formed with Si ion implanted source regions in a Mg ion implanted p-type base with N ion implanted termination regions. A maximum drain current of 115 mA/mm, maximum transconductance of 19 mS/mm at a drain voltage of 15 V, and a threshold voltage of 3.6 V were obtained for the fabricated DMOSFET with a gate length of 0.4 μm with an estimated p-type base Mg surface concentration of 5 × 10^18^ cm^−3^. The difference between calculated and measured V_th_s could be due to the activation ratio of ion-implanted Mg as well as Fermi level pinning and the interface state density. On-resistance of 9.3 mΩ·cm^2^ estimated from the linear region was also attained. Blocking voltage at off-state was 213 V. The fully ion implanted GaN DMOSFET is a promising candidate for future high-voltage and high-power applications.

## 1. Introduction

Wide-bandgap-based vertical power devices with normally-off operation have been developed in recent years [1,2,3,4,5,6,7,8]. The vertical devices are essential parts for power electronics in electric vehicles, data centers, smart grids, and renewable energy processes [9,10]. Silicon carbide (SiC) vertical MOSFETs are widely used in power applications. The early SiC power MOSFETs were vertical trench MOSFETs (UMOSFETs), in which the base and source regions were formed epitaxially, without the need for ion implantation [2]. One of the disadvantages of the trench MOSFETs is the problem with oxide breakdown at the trench corners. The planar double-implanted vertical MOSFETs (DMOSFETs) were developed to avoid critical electric field at the trench corners [11]. The p-type base and the n-type source regions are formed by successive ion implantation and high-temperature annealing procedures. Gallium nitride (GaN) is an ideally suitable material for applications in high-power, high-frequency, and high-temperature devices due to its remarkable properties [12]. Except for the applications of using GaN in photonics [13,14,15], the electric power devices with normally-off operation have progressed rapidly in recent years [5,7]. In conventional GaN technology, p-type and n-type layers are formed by impurity doping during epitaxial growth [16,17,18]. Thus, recent GaN vertical power transistors have trench gate structures and low-resistance source regions utilized two-dimensional electron gas (2DEG) produced by polarization charges at the hetero-interface [19]. Ion implantation is a widely used doping technology for Si and SiC MOSFETs but it has been difficult to form a p-type doping layer using ion implantation technology for GaN device fabrication process until recently. To obtain a high-quality p-type layer using ion implantation, an annealing procedure with temperatures higher than the epitaxial growth temperature of the GaN layer on a GaN or sapphire substrate is required. Though the formation of a p-type GaN layer and a p-n junction by Mg ion implantation have been reported [20,21,22], there have been a few reports about vertical devices fabricated in the Mg ion implanted layer [23]. In this paper we demonstrate GaN DMOSFETs with atomic layer deposition (ALD)-Al_2_O_3_ gate dielectric films fabricated on free-standing GaN substrates for the first time, by incorporating Si ion implanted regions into Mg ion implanted regions.

## 2. Double Ion Implantation into GaN

**Figure 1:**
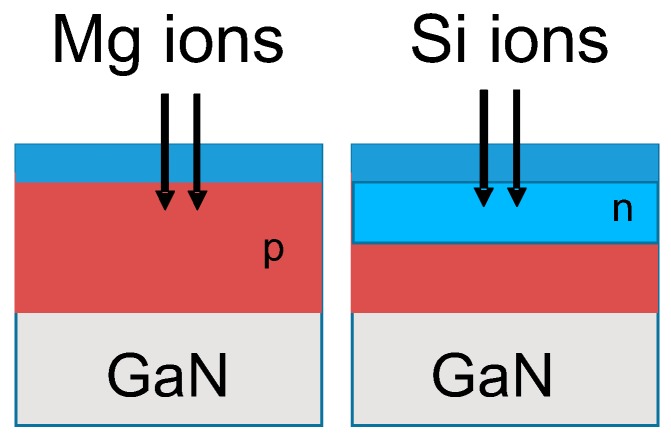
Mg ion implantation and double ion implantation of Mg and Si.

**Figure 2:**
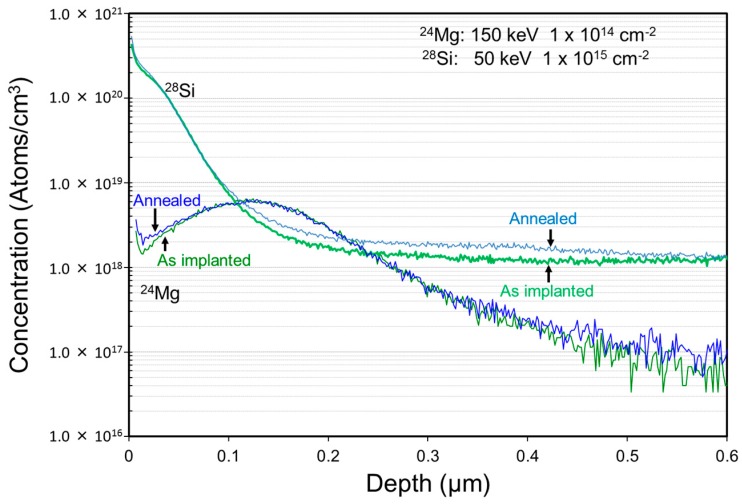
Ion-implanted Mg and Si profiles in a free-standing GaN substrate. Si background concentration in the substrate was about 2 × 10^18^ cm^−3^.

**Figure 3:**
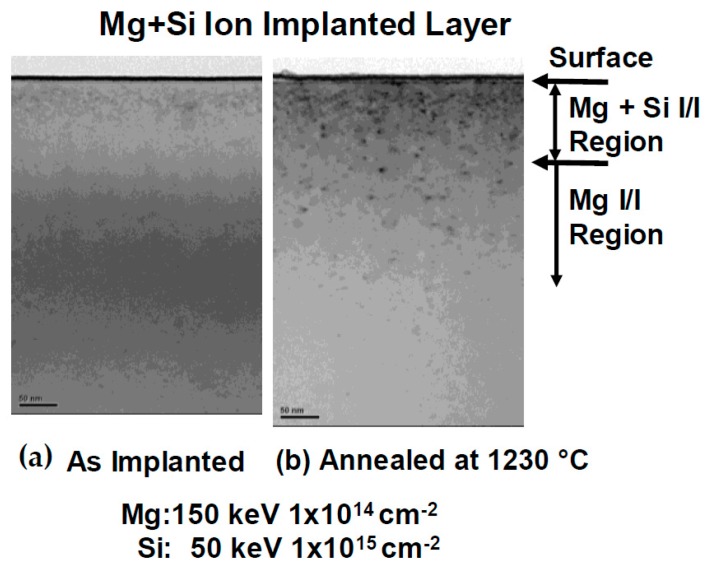
TEM images of double ion implanted layer of Mg and Si: (**a**) as-implanted, (**b**) annealed at 1230 °C.

Prior to device fabrication, the properties of damage recovery on Mg ion implanted p-type layers were investigated. Schematic cross sections of implanted layers are shown in Figure 1. Mg + Si ions were implanted into free-standing GaN substrates. After Mg ion implantation at an energy of 150 keV with a dose of 1 × 10^14^ cm^−2^, Si ions at an energy of 50 keV with a dose of 1 × 10^15^ cm^−2^ were then successively implanted, followed by annealing at 1230 °C for 1 min in N_2_ gas ambient. Implanted Mg and Si profiles measured by secondary ion mass spectrometry (SIMS, EAG Laboratories, Sunnyvale, CA, USA) in free-standing GaN substrate before/after annealing are shown in Figure 2. Mg profiles before/after annealing did not change. The background of Si concentration included in free-standing GaN substrate was about 2 × 10^18^ cm^−3^. The depth of the p-n junction fabricated by Si ion implantation in the Mg-doped p-type layer was estimated at 100 nm. Transmission electron microscope (TEM, EAG Laboratories, Sunnyvale, CA, USA) images of the Mg implanted layer and the double (Mg and Si) ion implanted layer are shown in Figure 3. Many defects were still present in the Si implanted layer after high-temperature annealing, but it seems that the defects that were more clearly seen after annealing in the Mg ion implanted layer were due to the localization of Mg atoms.

## 3. Device Structure and Fabrication

**Figure 4:**
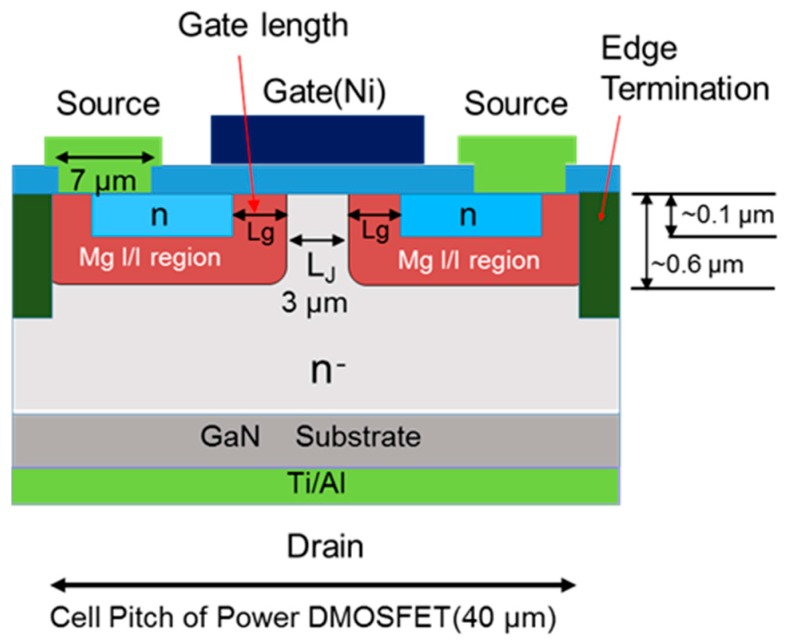
Schematic cross section of the fully ion implanted vertical GaN DMOSFET.

**Figure 5:**
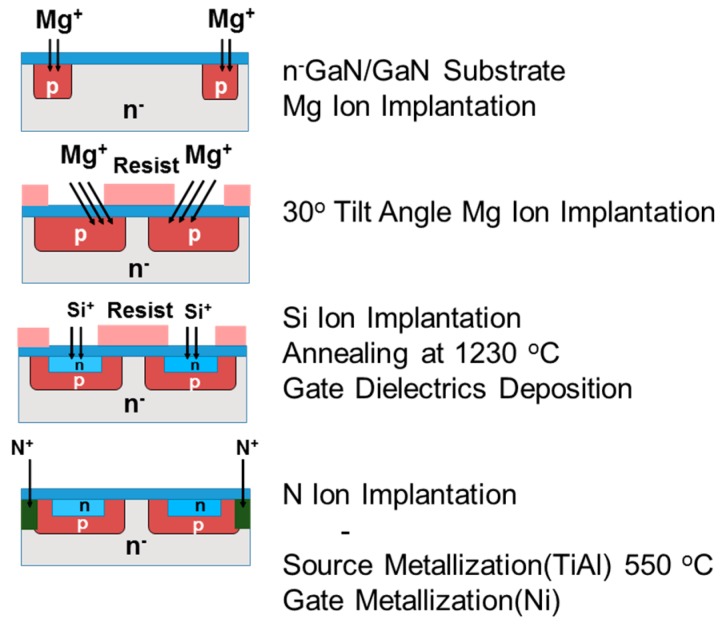
Fabrication process of the GaN DMOSFET by triple ion implantation.

**Figure 6:**
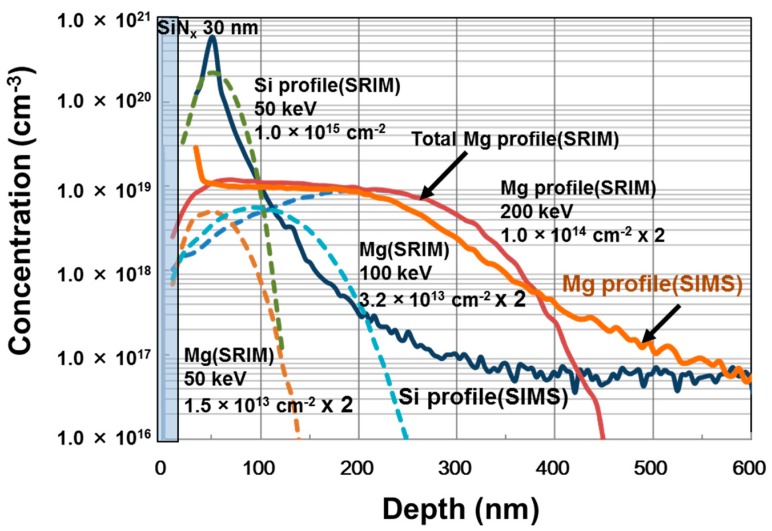
Ion-implanted Mg and Si profiles in a free-standing GaN substrate. Si background concentration in the substrate was about 5 × 10^16^ cm^−3^.

**Figure 7:**
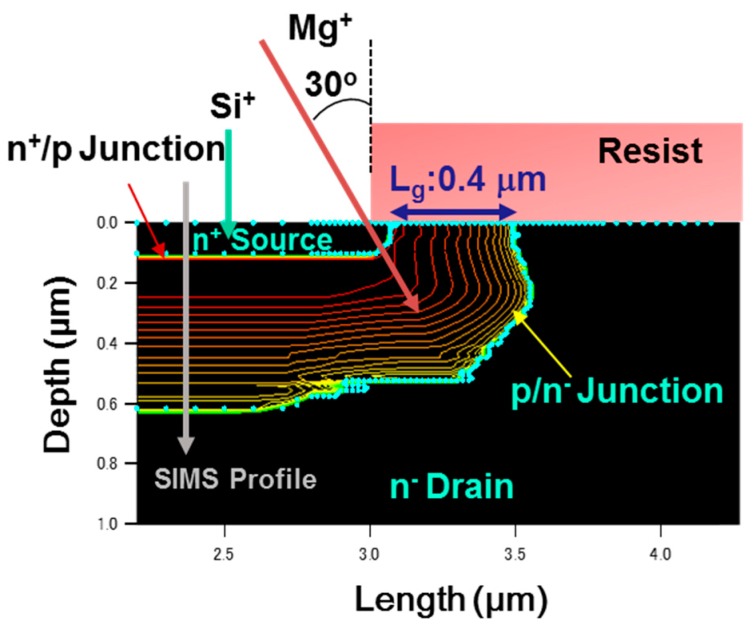
Simulated Mg surface concentration and gate length (L_g_) of the GaN DMOSFET fabricated by tilted Mg and Si ion implantation. The lateral expansion of Mg and Si profiles were simulated using the secondary ion mass spectrometry (SIMS) profiles.

**Figure 8:**
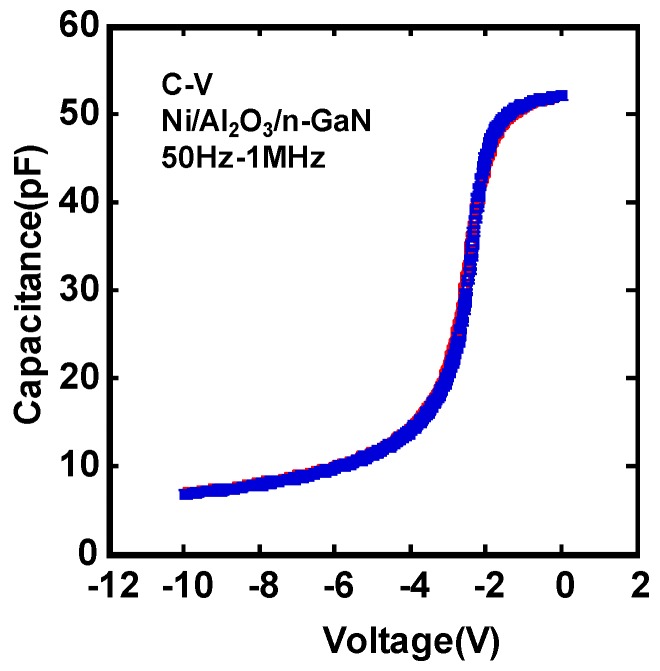
The C-V characteristics of Ni/Al_2_O_3_/n-GaN MOS capacitors measured from 50 Hz to 1 MHz.

A schematic cross section of the device structure of an ion-implanted GaN DMOSFET on a free-standing GaN substrate is shown in Figure 4. The channel regions were fabricated in the Mg ion-implanted layers and the gate length was self-alignedly defined by the difference in the depths between Mg and Si implanted regions. The fabrication process of the DMOSFET is illustrated in Figure 5. The GaN layer (5 μm) with a Si density of 5 × 10^16^ cm^−3^ was grown by metal-organic vapor phase epitaxy (MOVPE) on a free-standing GaN substrate with a low threading dislocation density of 10^6^ cm^−2^. Mg ions were implanted to form contact regions of the p-base regions at first. Mg ion implantation at a tilt angle of 30° was then carried out to form deep retrograde p-base regions in which channel and n-type source regions were formed. Mg ions were implanted for the left hand-side and right hand-side of the photoresist (OFPR-800, 2 μm-thick) mask region at three different energies of 200, 100, and 50 keV with doses of 1.0 × 10^14^, 3.2 × 10^13^, and 1.5 × 10^13^ cm^−2^ (single side total dose: 1.47 × 10^14^ cm^−2^) through 30-nm-thick SiN_x_ film, respectively. The junction field effect transistor (JFET) gap (L_J_), defined by the distance between two adjacent p-bases, was determined by the photoresist mask dimension. After Mg ion implantation, Si ions were successively implanted at an energy of 50 keV with a dose of 1 × 10^15^ cm^−2^ to form source regions. Then, the SiN_x_ film was removed and a 50 nm-thick SiN_x_ film was deposited again, followed by Mg and Si activation annealing at 1230 °C for 1 min in N_2_ gas ambient. N ions were then implanted to form edge termination regions [24] at an energy of 100 keV with a dose of 1.2 × 10^15^ cm^−2^. After the SiN_x_ film was removed, Al_2_O_3_ gate dielectric films of 45 nm were deposited by atomic layer deposition (ALD) at a temperature of 260 °C. Ohmic contacts were formed by depositing Ti/Al (50/300 nm) layers, followed by post metallization annealing at 550 °C for 1 min. Finally, gate electrodes were also formed by depositing Ni layers. Implanted Mg and Si profiles measured by SIMS after annealing are shown in Figure 6. The simulated impurity profiles of the implanted Mg and Si calculated by the stopping and range of ions in matter (SRIM) simulation are also shown. The channel regions were self-aligned to the left hand-side and right hand-side of the photoresist mask region during ion implantation to introduce the respective dopants, as shown in Figure 5. Lateral expansion of Mg and Si profiles were simulated using SIMS profiles. The channel length (L_g_) of 0.4 μm was determined by the difference in lateral extension of the Mg implanted p-base (p/n junction) and the Si implanted n-type source region (n^+^/p junction) at the surface after annealing, as shown in Figure 7. Mg surface concentration at the DMOSFET channel regions was also estimated as 5.0 × 10^18^ cm^−3^. The C-V curve of 45 nm Ni/ALD-Al_2_O_3_/n-GaN MOS capacitors measured at frequencies ranging from 50 Hz to 1 MHz is shown in Figure 8. Frequency dispersion was not observed in this frequency range. The dielectric constant of 8.5 and MOSFET capacitance of 1.71 × 10^−7^ F/cm^2^ were measured.

## 4. Device Performances and Discussion

**Figure 9:**
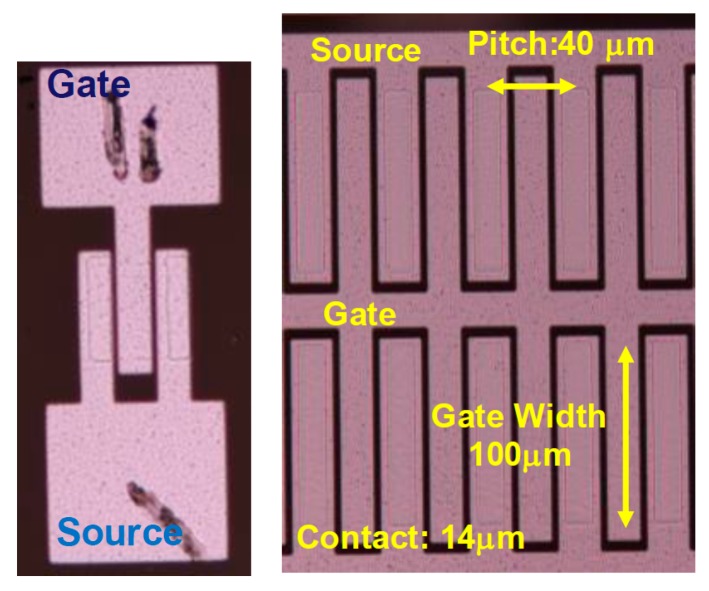
Plane view of the fabricated GaN DMOSFET with an L_g_ of 0.4 μm and W_g_ of 50 μm. The cell pitch of the power DMOSFET was 40 μm.

Plane view of the fabricated single GaN DMOSFET with a gate length of 0.4 μm, JFET gap (L_J_) of 3 μm, and gate width of 50 μm is shown in Figure 9. The cell pitch of the power DMOSFET was 40 μm for the gate width of 100 μm (Figure 9, right side). A low sheet resistance of 139 Ω/square and a contact resistance as low as 0.53 Ω·mm for the n^+^ source regions were obtained [25]. Ohmic contact to the surface of the Mg ion-implanted regions could not be formed, because the carrier concentration of the Mg ion-implanted contact layer was estimated to be below 1 × 10^18^ cm^−3^ due to an Mg acceptor level as deep as 200 meV [26]. Therefore, it is considered that Mg ion implanted p-base regions were kept at a floating potential or connected as a Schottky contact to the source electrodes.

**Figure 10:**
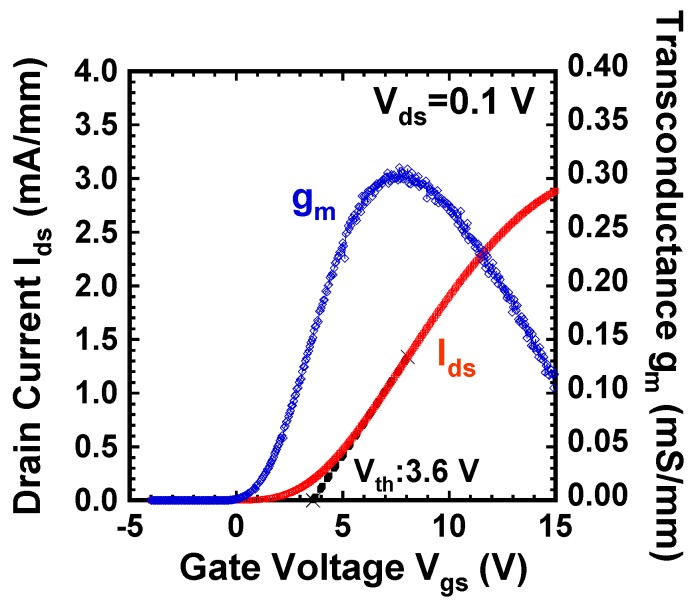
I_ds_-V_gs_ and g_m_-V_gs_ characteristics at a linear region of the DMOSFET.

**Figure 11:**
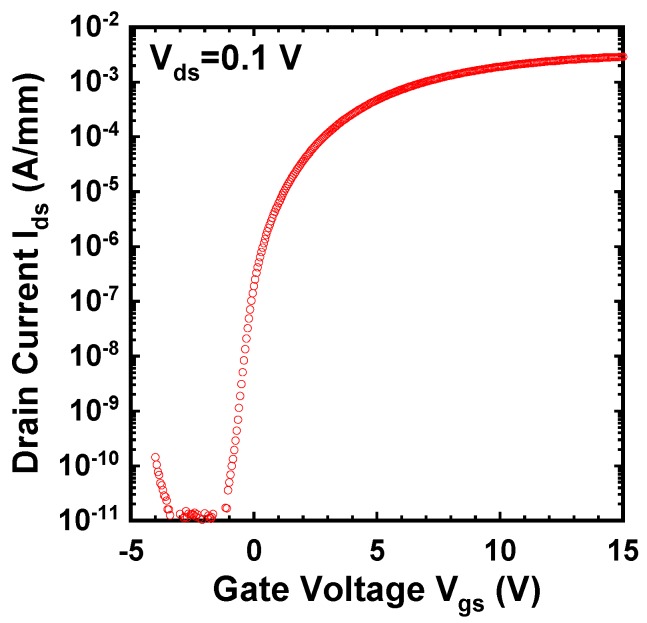
Subthreshold characteristics of the DMOSFET. D_it_ estimated from subthreshold slope of 264 mV/dec was 2.1 × 10^12^ cm^−2^·eV^−1^.

**Figure 12:**
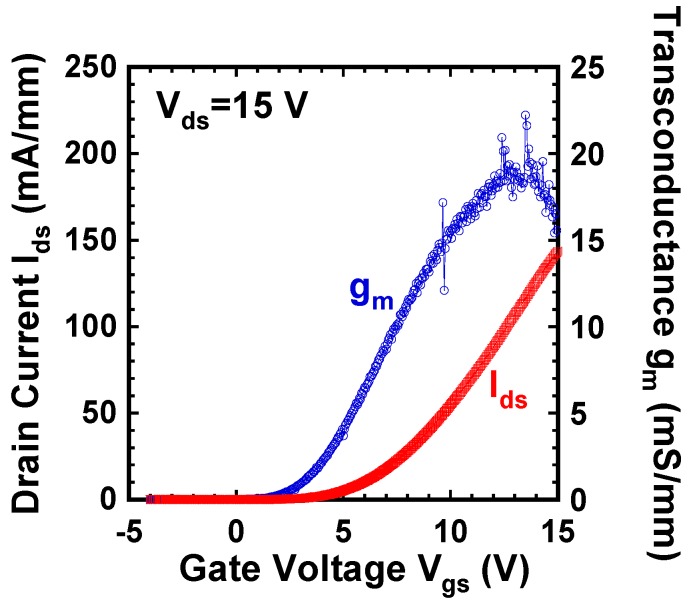
I_ds_-V_gs_ and g_m_-V_gs_ characteristics of the DMOSFET at V_ds_ = 15 V.

**Figure 13:**
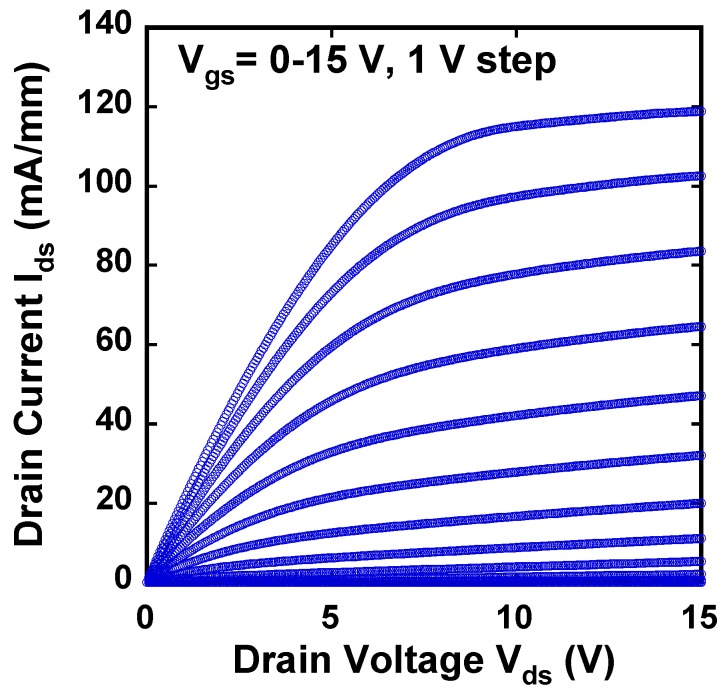
I_ds_-V_ds_ characteristics of the DMOSFET. On-resistance of 46.4 Ω·mm was measured at V_gs_ = 15 V and V_ds_ = 0.5 V.

**Figure 14:**
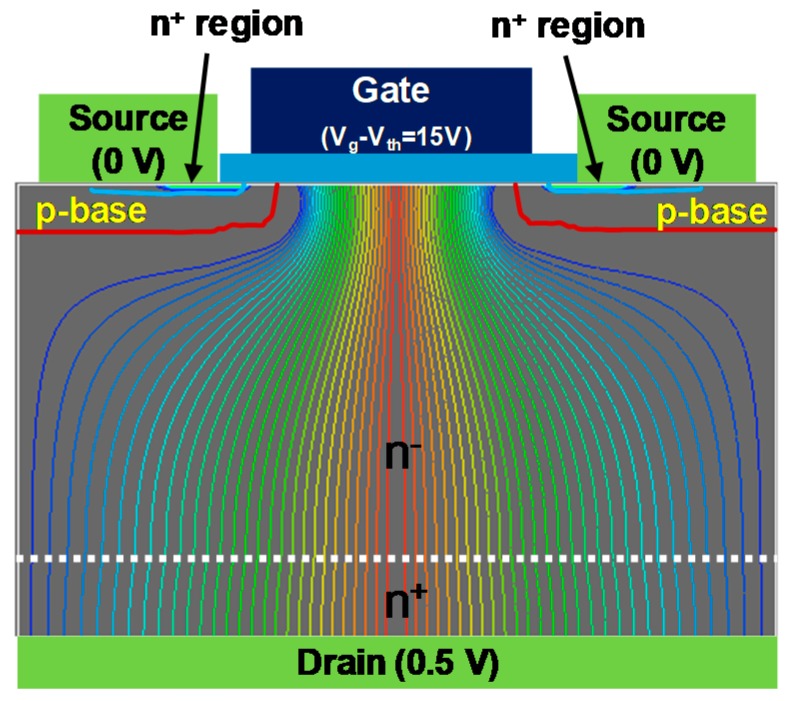
Simulated linear region electron current flow of the GaN DMOSFET with an L_J_ of 3.0 μm at V_gs_ = 15 V and V_ds_ = 0.5 V. In saturation regions, the electron current flows without tightening in an L_J_ above 3 μm.

**Figure 15:**
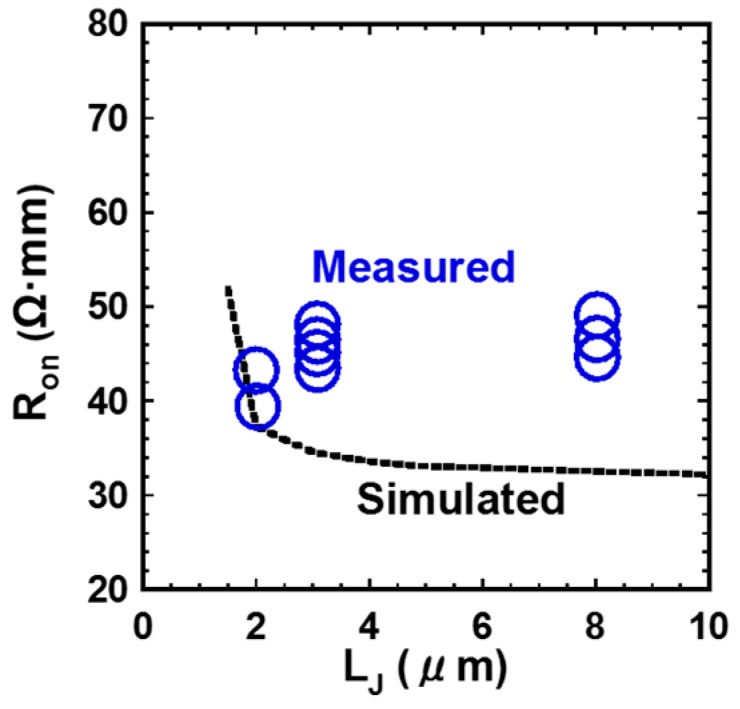
On-resistance as a function of JFET gap (L_J_).

**Figure 16:**
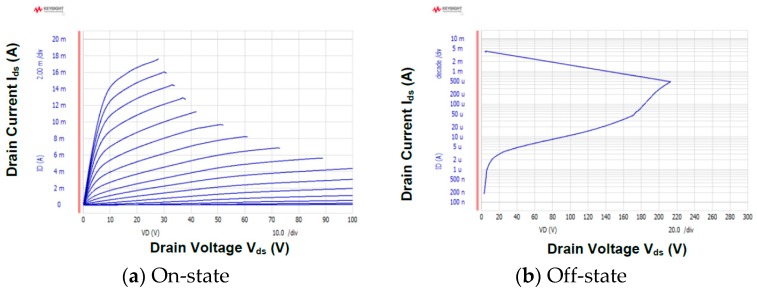
(**a**) On-state and (**b**) off-state I_ds_-V_ds_ characteristics of the DMOSFET (L_g_ = 0.4 μm and W_g_ = 100 μm). The blocking voltage at off-state was 213 V.

Figure 10 shows the I_ds_-V_gs_ and g_m_-V_gs_ characteristics of the fabricated GaN DMOSFET at a drain voltage of 0.1 V. The V_th_ of the DMOSFET obtained from extrapolation of linear portion of I_ds_-V_gs_ characteristics using the extrapolation in the linear region (ELR) method [27] was about 3.6 V. The calculated V_th_ of 16 V from the flat band without surface state and trap densities was obtained from the equation, $V_{th}=2\psi_{B}+\sqrt{2\varepsilon_{GaN}qN_{A}(2\psi_{B}})/C_{g}$ [28], where $\psi_{B}$ is the Fermi level from the intrinsic Fermi level in the Mg-doped layer, $\varepsilon_{GaN}$ is the dielectric constant of GaN, q is the unit electronic charge, $N_{A}$ is the acceptor concentration, and $C_{g}$ is gate capacitance. The surface Mg concentration of 5 × 10^18^ cm^−3^, $\psi_{B}$ of 1.66 eV, and gate capacitance of 1.7 × 10^−7^ F/cm^2^ were used for the V_th_ calculation. The difference between calculated and measured V_th_s could be due to Fermi level pinning at the p-GaN surface [29,30], the D_it_, and the activation ratio of ion-implanted Mg in the channel region. The Fermi level of the p-GaN surface at the Mg concentration of 1.3 × 10^18^ cm^−3^ was pinned at about 2.4 eV above the E_v_ and about 1.0 eV below E_c_ [30]. V_th_ was reduced by both 2.4 V for Fermi level pinning and 1.94 V for the D_it_ calculated from the subthreshold characteristics described below. The acceptor concentration depends on Mg atoms substituting for Ga sites in the GaN lattice, and is determined by the activation ratio of implanted Mg atoms by annealing condition and Mg doses [22]. When the activation ratio of 20% and the acceptor concentration of 1 × 10^18^ cm^−3^ instead of 5 × 10^18^ cm^−3^ were used, a V_th_ of 8.9 V was calculated and the influence on V_th_ reduction was predicted to be dominant. Therefore, one of the major reasons for the V_th_ difference is considered to be acceptor concentration. The field effect mobility of 7.1 cm^2^/(V·s) was extracted by g_m_-V_gs_ characteristics. This value is close to that of the GaN MOSFET fabricated in a p-type epilayer grown on sapphire substrate [31]. Though the V_th_ shifted in a negative direction, the mobility increased up to 11.0 cm^2^/(V·s) as total implanted Mg doses decreased to 3.65 × 10^13^ cm^−2^. The crystalline quality of the Mg ion-implanted GaN with higher mobility would be restored by higher-temperature annealing [32,33]. Subthreshold characteristics of the device at a drain voltage of 0.1 V are shown in Figure 11. Interface state density (D_it_) estimated from a subthreshold slope of 264 mV/dec [34] was 2.1 × 10^12^ cm^−2^·eV^−1^, which was in good agreement with recessed gate GaN-FETs with ALD-Al_2_O_3_ gate dielectrics [35]. I_OFF_ and I_ON_ were measured at V_gs_ = 0 V and V_gs_ = 3.6 V, respectively. The I_ON_/I_OFF_ ratio was about 1 × 10^3^ (V_ds_ = 0.1 V, V_on_ − V_off_ = 3.6 V). I_dsm_ and g_mmax_ at V_gs_ of 13.5 V were 115 mA/mm and 19 mS/mm at V_ds_ of 15 V, respectively, as shown in Figure 12. Figure 13 shows the I_ds_-V_ds_ characteristics of the DMOSFET. The specific R_on_ obtained from the linear region at V_ds_ of 0.5 V and V_gs_ of 15 V was 46.4 Ω·mm, which was estimated to be equivalent to 9.3 mΩ·cm^2^. The simulated electron current flow of the DMOSFET with an L_J_ of 3 μm is shown in Figure 14. The electron current flow spread around the Mg-implanted p-type regions and the JFET component resistance in on-resistance (R_on_) seemed to become dominant when L_J_ was below 2 μm. Figure 15 shows the L_J_ dependence of the measured R_on_. The measured R_on_s ranged from 40 to 50 Ω·mm, which were nearly in good agreement with numerically simulated results. Lower R_on_ could be achieved by reducing the sheet resistivity of the n-type epitaxial layer and the cell pitch of the DMOSFET. Figure 16 shows the on-state and off-state pulsed I_ds_-V_ds_ characteristics of the fabricated GaN DMOSFET measured at pulse width/period of 5/120 ms. Blocking voltage at the off-state was 213 V, which is lower than the expected value for the epitaxial layer thickness of 5 μm. It seems that blocking voltage was limited by the source-drain electric field at the Mg-implanted p-base peripheral cylindrical regions and the gate-drain electric field at the N-implanted edge termination regions. Higher blocking voltage would be attained by fabricating deeper Mg ion implanted regions.

The self-aligned GaN DMOSFETs fabricated by tilted angle Mg and Si ion implantations were demonstrated. These results exhibited that the n-type regions were successfully formed in the Mg ion-implanted p-base layers, and an innovative performance was achieved. Additionally, these indicate a definite availability of normally-off GaN DMOSFET for power device applications. Further improvement of the V_th_ control and blocking voltage can be expected by refining Mg ion implantation, activation annealing procedures, surface treatment of the deposition of gate dielectrics, and optimization of device structure using field plate electrodes. Moreover, further miniaturization of the device layout will enable a much lower R_ON_ to be obtained.

## 5. Conclusions

We have demonstrated a normally-off, self-aligned GaN DMOSFET with a gate length of 0.4 μm fabricated by the double ion implantation of Mg and Si for the first time. V_th_ obtained from extrapolation of a linear portion of g_m_ was about 3.6 V. I_dsm_ and g_mmax_ at a drain voltage of 15 V for the DMOSFET was 115 mA/mm and 19 mS/mm, respectively. The blocking voltage at off-state was 213 V. R_on_ estimated from the linear region was 9.3 mΩ·cm^2^. The difference between calculated and measured V_th_s could be due to the activation ratio of ion-implanted Mg as well as Fermi level pinning and the interface state density. High-performance normally-off vertical GaN DMOSFETs can be achieved by further improvement of the double ion implantation procedure, especially the development of higher-temperature annealing processes.
